# Integrated management of charcoal rot disease in susceptible genotypes of mungbean with soil application of micronutrient zinc and green manure (prickly sesban)

**DOI:** 10.3389/fmicb.2022.899224

**Published:** 2022-07-25

**Authors:** Amna Shoaib, Kashif Ali Khan, Zoia Arshad Awan, Basit Latief Jan, Prashant Kaushik

**Affiliations:** ^1^Department of Plant Pathology, Faculty of Agricultural Sciences, University of the Punjab, Lahore, Pakistan; ^2^Department of Clinical Pharmacy, College of Pharmacy, King Saud University, Riyadh, Saudi Arabia; ^3^Instituto de Conservación y Mejora de la Agrodiversidad Valenciana, Universitat Politècnica de València, Valencia, Spain

**Keywords:** antioxidant enzymes, systemic resistance, reactive oxygen species, green manure, mungbean, charcoal rot, zinc

## Abstract

Charcoal rot disease is incited by the soil-borne fungus *Macrophomina phaseolina* (Tassi). Goid is a challenging disease due to long-term persistence of fungus sclerotia in the soil. This study assessed the potential of zinc (Zn: 1.25, 2.44, and 5 mg/kg) and green manure (GM: 1 and 2%) in solitary and bilateral combinations to alleviate infection stress incited by *M. phaseolina* on disease, growth, physiology, and yield attributes in mungbean. A completely randomized design experiment was conducted in potted soil, artificially inoculated with the pathogen, and sown with surface-sterilized seeds of mungbean genotypes (susceptible: MNUYT-107 and highly susceptible: MNUYT-105). Concealment of plant resistance by *M. phaseolina* in both genotypes resulted in 53–55% disease incidence and 40–50% plant mortality, which contributed in causing a significant reduction of 30–90% in attributes of growth, biomass, yield, photosynthetic pigment, and total protein content with an imbalance of production of antioxidant enzymes (polyphenol oxidase, superoxide dismutase, catalase, and peroxidase). Soil application with Zn-based fertilizer (ZnSO_4_: 33%) in combination with GM significantly managed up to 80% of the charcoal rot disease, hence improving growth (50–100%) and physiochemical (30–100%) attributes and sustainably enhancing grain average yield (300–600%), biological yield (100–200%), and harvest index (100–200%) in mungbean plants. The heat map and principal component analyses based on 19 measured attributes with 16 treatments separated Zn (2.44 or 5 mg/kg) combined with 2% GM as the best treatments for alleviating charcoal rot disease stress by improving growth, yield, and biological attributes to an extent to profitable farming in terms of harvest index (HI) and benefit-cost ratio (BCR).

## Introduction

Mungbean is a very attractive early-maturing legume for farmers in South Asia, including Pakistan, because it yields modestly even under harsh climatic conditions. However, the availability of low-quality seeds, high labor costs, and overall knowledge gap about mungbean’s effect on soil fertility are among the common constraints in mungbean cultivation in South Asia (Rani et al., 2018). In addition to these constraints, pests and diseases also affected mungbean yield in Pakistan, with charcoal rot caused by *Macrophomina phaseolina* (Tassi) Goid known to significantly reduce crop yield (Khan et al., 2018; Shoaib et al., 2022). *M. phaseolina* is one of the most destructive seed and soil-borne fungi present all over the world. It causes charcoal rot disease in more than 750 plant species, including pulses, especially in arid to tropical regions. The pathogen produces heat-tolerant sclerotia, which is released in clusters on the soil surface to colonize the living and dead tissues of the host. The sclerotia can survive in soil and root debrides for a long time (2–15 years) under field conditions (Nazli et al., 2020). *M. phaseolina* can attack the plants at any growth stage and develop irregular dark lesions on the hypocotyls and epicotyls that extend to the cotyledons. In mature plants, pathogen infestation blocks the xylem bundles and causes the death of the host (Khan et al., 2018). Under high temperatures (30–35°C) and low soil moisture (below 60%), the infection may result in a yield loss of up to 100%, impacting the incomes of the farmers (Marquez et al., 2021).

The application of chemical fungicides (e.g., captan and quintozene) may provide a solution against charcoal rot disease. However, the high cost of chemical fungicides as well as their potentially harmful effects on all living beings destroy the theme of sustainable agriculture (Meyer and Hausbeck, 2012). The application of micronutrient zinc (Zn) can give a revolutionary resolution to sustainable agriculture in an environment-friendly way against devastating charcoal rot in mungbean by tempting resistance in plants (Shoaib et al., 2021, 2022). Zn also plays a pivotal role in the metabolism of the plant, and nearly half of the crop-growing areas around the world have low plant-available Zn in their soils, with its deficiency estimated to reduce the Gross Domestic Product (GDP) of some developing countries by 2–11% (Darnton-Hill et al., 2005).

The role of Zn as a cofactor for the initiation of numerous enzymes mandatory for redox reactions and synthesis of proteins in plants has been well-appreciated, and Zn essentiality in the formation of auxin, chlorophyll, and some carbohydrates is also well-recognized (Cabot et al., 2019). It plays an essential role in repairing photosystem II, regulating plant growth and reproduction, lipid and nucleic acid metabolism, gene expression and regulation, signal transduction *via* mitogen-activated protein kinases, and maintenance of membrane integrity (Khan et al., 2021). Along this line, Zn acts as the first line of defense and strengthens the plant’s physical and mechanical properties, hence enhancing plant resistance against diseases (Cabot et al., 2019). Zn-efficient genotypes have been found to impair plant production by reducing pathogen susceptibility (Shoaib et al., 2020, 2022). While the soil application of Zn fertilizers is an effective way to enhance plant resistance against diseases and sustain crop yield (Cakmak, 2008). It is stated that the bioavailability of Zn from the soil is increased by the action of organic acids exuded by roots, which saturate the lower tissues of the stem (da Cruz et al., 2019). This, therefore, can induce systemic resistance in plants and antifungal action against the soil-borne pathogens (Awan et al., 2019; Shoaib et al., 2020).

Furthermore, soil application with Zn-based fertilizers has been reported to alleviate biotic stress and increase productivity by boosting the activity of antioxidant enzymes [catalase (CAT), peroxidase (POX), superoxide dismutase (SOD), and polyphenol oxidase (PPO)] against oxidative stress induced by reactive oxygen species (ROS) (Khan et al., 2018; Ahmad et al., 2019, 2020; Shoaib et al., 2020; Mansoor et al., 2022). Quaglia et al. (2021) findings revealed that Zn phosphate induced morphological and biochemical plant defense responses in tomatoes and direct antimicrobial activity against *Pseudomonas syringae* pv. tomato. Awan et al. (2019, 2022) explored that soil application with Zn-based fertilizer (ZnSO_4_: 2.5 and 5 mg/kg) can manage up to 70% of early blight disease, hence improving growth, total phenolic content, and photosynthetic pigment in tomato plants by altering the activities of antioxidant enzymes (e.g., CAT, POX, PAL, and PPO) in the plant. The protective role of Zn against ROS has also been linked to reducing the production of membrane-bounded NADPH oxidase (Shoaib et al., 2021). In many other studies, Zn has been recognized as a potential inhibitor of fungal pathogens like *Fusarium graminearum*, *Penicillium citrinum*, *Aspergillus flavus* (Savi et al., 2013), *Alternaria alternata* (Shoaib et al., 2021), and *M. phaseolina* (Shoaib et al., 2020, 2022). In these studies, the antifungal potential of Zn has been linked with disruption in the internal hydrostatic pressure of the fungal cell along with suppression in morpho-growth characteristics. Literature also revealed ZnSO_4_ as an easily available and economically feasible source of Zn due to its higher solubility compared to oxides and carbonates (da Cruz et al., 2019). Thus, the use of Zn-based fertilizers (e.g., ZnSO_4_) can ensure higher productivity (Liu et al., 2020; Zulfiqar et al., 2020) and could be an alternative disease management strategy as durable, economic, and self-maintaining as compared to chemical fungicides (Khan et al., 2018).

Over and above, the plant defense system can further be strengthened by using Zn in combination with organic manure (Cakmak, 2012; Awan et al., 2019). It has been suggested that using the best combination of an appropriate proportion of inorganic fertilizers with organic manure can improve plant nutrient acquisition traits (Shen et al., 2016). Studies indicate that the combined applications of Zn fertilizer together with organic materials are particularly effective in aiding Zn uptake by roots and improving the enormity of Zn deficiency (Cakmak, 2008). Green manure (GM) is an economical and accessible organic soil amendment, which helps in improving soil’s physical properties (e.g., degradation, aeration, water retention, and infiltration) by decreasing soil erosion and leaching losses, adding a considerable amount of carbon, nitrogen, and secondary metabolites, alleviating the supply of available plant nutrients, and promoting microbiological properties. GM crops also support in the suppression of weeds, pests, and disease problems (Rani et al., 2021). Various antifungal compounds like ammonia and nitrous acid are reported to be produced by the decomposition of GM, providing supplementary plant protection by inducing systemic resistance in plants (Li et al., 2016). *Sesbania bispinosa* (prickly sesban or canicha) is a tropical valuable legume for its fast growth and high biomass amount, hence commonly used as a green manure crop to add low-cost nitrogen inputs to crop production systems in order to achieve a higher income level in a sustainable agriculture manner (Al-Masri and Mardini, 2008). Although many researchers’ findings testify to the disease managing potential of Zn or GM, literature is scanty on the combined application of Zn and GM in managing plant diseases. Therefore, the current investigation was aimed to check the charcoal rot disease managing potential of Zn and GM (*S. bispinosa*) and their effects on growth, yield, and antioxidants of mungbean in Zn-deficient soils of Bhakkar, Punjab, Pakistan.

## Materials and methods

### Trial description and layout

The soil was collected from mungbean growing areas, 13-TDA 31° 38′ 0″ North, 71° 4′ 0″ East District Bhakkar, Punjab, and the experiments were carried out in the summer season (May–July, average temperature: 40°C ± 5°C and average relative humidity: 50 ± 5%). The sterilization of the sandy-loam soil (0.43% organic matter, 2.07 dS m^–1^ electrical conductivity, 8.1 pH, and 32% saturation) was achieved by fumigation with formalin-dipped cotton plugs buried in many places in the heap of soil (Khan et al., 2016). The sterilized soil was filled in the pots (7″ × 6″ h × w, 5 kg pot^–1^), artificially inoculated by 50-ml cultural suspension of *M. phaseolina* (FCBP 0751), and left for 7 days for fungal colonization. Then, fully decomposed *S. bispinosa* (GM: 1 and 2%) was mixed thoroughly in the potting soil. Seeds of highly susceptible (MNUYT-7) and moderately susceptible (MNUYT-105) genotypes of mungbean were collected from the Arid Zone Research Institute, Bhakkar, and Ayub Agricultural Research Institute, Faisalabad, Pakistan (Khan et al., 2016). The seeds were surface sterilized in 1% Clorox solution and were sown (7 seeds/pot) in the pathogen-inoculated soil. Three doses (1.25, 2.44, and 5 mg/kg) of Zn fertilizer were applied separately as basal 30 days after seed germination. Zn fertilizer [ZnSO_4_. H_2_O (33%)] was obtained, with the brand name Zingro, from registered dealers of Engro Fertilizers Limited, Pakistan. All pots were arranged randomly, kept under the same conditions, and watered regularly. There were 16 treatments for each genotype (Table 1). Each treatment was replicated thrice, and the experiment was intended for 70 days.

**TABLE 1:** Treatments designed for the current experiment.

Zinc dose (mg/kg)	−ve Control	+ve Control (*M. phaseolina*)	GM + *M. Phaseolina*	
			1%	2%
0	T_1_	T_2_	T_3_	T_4_
1.25	T_5_	T_6_	T_7_	T_8_
2.44	T_9_	T_10_	T_11_	T_l2_
5.00	T_l3_	T_l4_	T_l5_	T_16_

T_1_, Negative control; T_2_, Positive control [M. phaseolina(MP)]; T_3_, 1% GM + MP; T_4_, 2% GM + MP; T_5_, Zn (1.25 mg/kg); T_6_, Zn (1.25 mg/kg) + MP; T_7_, 1% GM + MP + Zn (1.25 mg/kg); T_8_, 2% GM + MP + Zn (1.25 mg/kg); T_9_, Zn (2.44 mg/kg); T_10_, Zn (2.44 mg/kg) + MP; T_11_, 1% GM + MP + Zn (2.44 mg/kg); T_12_, 2% GM + MP + Zn (2.44 mg/kg); T_13_, Zn (5 mg/kg); T_14_, Zn (5 mg/kg) + MP; T_15_, 1% GM + MP +Zn (5 mg/kg); T_16_, 2% GM + MP + Zn (5 mg/kg).

### Disease measurement

Data concerning disease pressure in terms of disease incidence and plant morality was checked on the 70th day after seed sowing (DAS) (Cohen et al., 2000).


$$\begin{array}{c} \mathrm{Disease}\mathrm{incidence}\left(\%\right)=\left(\mathrm{Number}\mathrm{of}\mathrm{infected}\mathrm{plants}\right)/\\ \;\left(\mathrm{Total}\mathrm{number}\mathrm{of}\mathrm{plants}\right)\times 100 \end{array}$$

$$\begin{array}{c} \mathrm{Plant}\mathrm{mortality}\left(\%\right)=\left(\mathrm{Number}\mathrm{of}\mathrm{dead}\mathrm{plants}\right)/\\ \;\left(\mathrm{Total}\mathrm{number}\mathrm{of}\mathrm{plants}\right)\times 100 \end{array}$$

### Photosynthetic pigment and antioxidants measurement

Total chlorophyll content, carotenoids, total protein content, and activity of SOD, CAT, POX, and PPO were carried out according to protocols described previously from the triplicate samples of leaves collected from each replicate at 35 days after sowing (DAS).

Total chlorophyll content was estimated in the leaf extract prepared in 80% ethanol, centrifuged at 10,000 rpm, and measured for chlorophyll a (645 nm), chlorophyll b (663 nm), and carotenoids (470 nm) on Spectro UV–vis double PC (Model UVD-2950). Total chlorophyll content and carotenoids were calculated against blank (80% ethanol only) using the formula specified by Lichtenthaler and Wellburn (1983).


Chlorophylla(mg/gFW)=[0.0127(OD 663)-0.00269(OD 645)(V/W)]

Chlorophyllb(mg/gFW)=[0.0229(OD 645)-0.00468(OD 663)(V/W)]

$$\begin{array}{c} \mathrm{Total}\mathrm{chlorophyll}\left(\mathrm{mg}/g\mathrm{FW}\right)=[\left(20.2\times \mathrm{OD645}\right)\\ +\left(8.02\times \mathrm{OD663}\right)\left(\frac{V}{1,000\times W}\right)] \end{array}$$

$$\begin{array}{c} \text{Carotenoids}=[1,000\mathrm{A470}-3.27\left(\mathrm{chlorophyll}a\right)\\ -104\left(\mathrm{chlorophyll}b\right)]/229 \end{array}$$

For the estimation of total protein content, the leaf sample (0.1 g) was crushed in 1 ml of phosphate buffer (0.1 M, pH 7.5) in a pre-chilled pestle and mortar in liquid nitrogen, centrifuged at 12,000 rpm for 15 min, and the supernatant (0.1 ml) was diluted with distilled water to make a final volume of 1 ml. The reaction mixture was prepared by adding reagent C [reagent A (0.2% NaOH and 2% Na_2_CO_3_), and reagent B (0.5% CuSO_4_ in 1% of KNaC_4_H_4_O_6_⋅4H_2_O) in a 50:1 ratio] followed by the addition of folin phenol reagent (0.1 ml). The total protein content of the reaction mixture was measured at 650 nm against the standard curve of bovine serum albumin (Lowry et al., 1951).

For the SOD assay, the sample was prepared by adding 30 mM methionine, 0.07 mM nitroblue tetrazolium (NBT), 0.1 mM EDTA, 0.002 mM riboflavin, and 0.1 ml enzyme extract. The control set contained the same reagents except for enzyme extract. Experimental and control sets were placed in light for 15 min, whereas blanks were placed in the dark. The absorbance for the activity of SOD was calculated at 560 nm (Maral et al., 1977).

The reaction mixture for the CAT activity contained 1 ml of enzyme extract, 1 ml of 0.01 M H_2_O_2_, and 1 ml of 0.1 M phosphate buffer. After incubation (5 min at 20°C), the reaction was stopped by adding 10 ml of 1% H_2_SO_4_. The mixture was titrated against 0.005 N, KMNO_4_ until pinkish color appeared (Maehly, 1954).

For the estimation of the POX, the enzyme extract (0.5 ml) was mixed with 1 ml of phosphate buffer (0.1 M), 1 ml of pyrogallol (0.01 M), and 1 ml of H_2_O_2_ (0.05 M). After incubation (5 min at 25°C), the reaction was stopped by adding 1 ml of H_2_SO_4_ (2.5 N), and the absorbance of the samples was immediately calculated at 420 nm (Piotrowska et al., 2010).

The PPO activity was estimated in the reaction mixture made of enzyme extract (0.1 ml), sodium phosphate buffer (0.1 M, pH 7), and 0.2 ml of catechol (0.01 M). The PPO activity was determined at 30 s interval for three times at 495 nm against a blank sample (Mayer et al., 1966).

### Biophysical growth and yield measurements

The growth parameters (plant tallness, fresh, and dry weight) and yield attributes, i.e., number of fully matured pods, grain yield per plant, 100-grain weight, and harvesting index percentage data were collected at final harvest (70 DAS) using the following equation:


Harvestindex(%)=(Grainyield)/(Biologicalyield)×100

### Statistical analysis

The Shapiro–Wilk normality test was used to check the collected data for normality in terms of morphological and biochemical attributes. A least significant difference test at *p* ≤ 0.05 was analyzed using the Statistix 8.1 software to assess the separate and interactive impacts of the applied treatments presented and interpreted in the manuscript. Two-way factorial ANOVA was used to measure the significant difference in the data. JMP^®^ (version 15.0, SAS Institute Inc., Cary, NC, United States, 1989–2019) was used to perform principal components analysis (PCA) and to construct a heat map.

## Results

Generally, all treatments significantly alleviated disease pressure (disease incidence, DI; plant mortality, PM), altered biochemical traits, and improved the biophysical attributes (vegetative and reproductive) in both genotypes of the mungbean as compared to their respective positive controls, but the combined effect of Zn (zinc) + GM (green manure) was more effective than their single effect.

### Disease pressure

**FIGURE 1:**
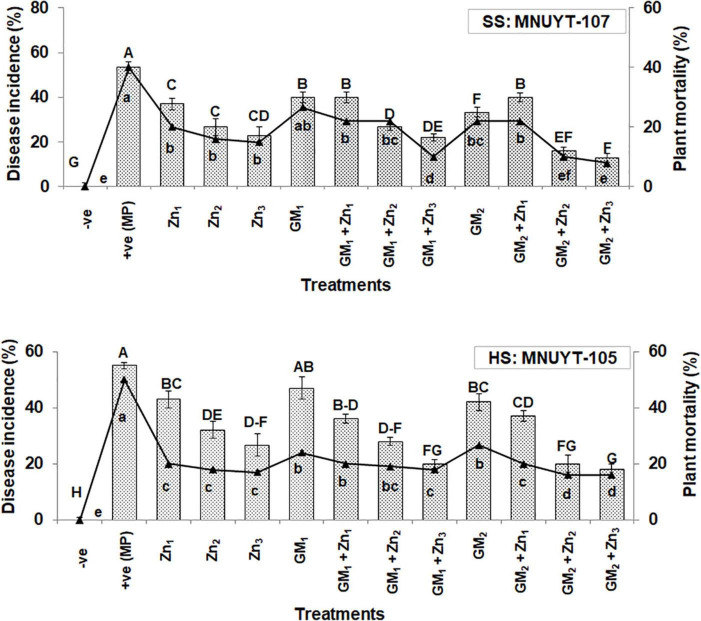
Effect of soil application with zinc (Zn) and green manure (GM) on charcoal rot disease incidence (%) and plant mortality (%) in susceptible (SS: MNUYT-107) and highly susceptible (HS: MNUYT-105) genotypes of mungbean after 70 days of sowing. –ve control (without pathogen, Zn or GM); +ve control [*M. phaseolina* (MP)]; Zn_1_ (1.25 mg/kg); Zn_2_ (2.44 mg/kg); Zn_3_ (5 mg/kg); GM_1_ (1%); and GM_2_ (2%). Vertical bars show standard errors of means of three replicates. Means with the same letters are not significantly different at *p* < 0.05 according to Fisher’s least significant difference (LSD) test.

Positive control [*M. phaseolina* only (MP)] treatments of susceptible genotype (MNUYT-107) showed the greatest disease pressure (DI: 53%; PM: 40%), which decreased significantly to 40% with 1% GM and 30% with 2% GM. The effect of Zn was dose-dependent, hence the minimum disease (DI: 23%; PM: 15%) was noticed with the application of 5 mg/kg of Zn, which was statically at par with effect of medium dose (2.44 mg/kg). Moreover, the disease reached a minimum (DI: 15%; PM: 10%) with all treatments containing medium or higher levels of Zn combined with 2% GM (Figure 1). Likewise, in the highly susceptible genotype (MNUYT-105), the maximum disease pressure (DI: 55%; PM: 50%) in positive control was reduced to the minimum (DI: 20%; PM: 16%) with the application of medium or higher doses of Zn combined with 2% GM as compared to remaining treatments (Figure 1).

### Biophysical growth attributes

**FIGURE 2:**
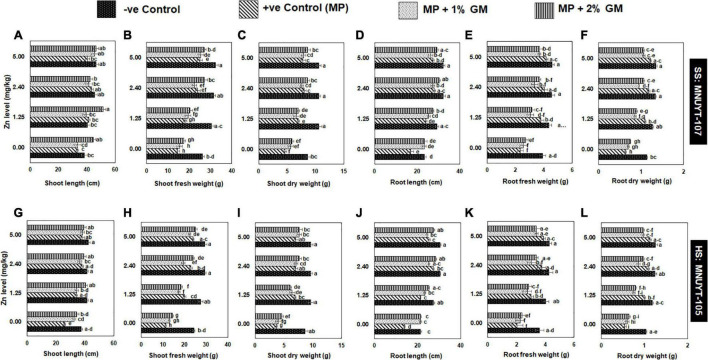
(A–L) Effect of soil application with zinc (Zn) and green manure (GM) on vegetative growth attributes in susceptible (SS: MNUYT-107) and highly susceptible (HS: MNUYT-105) genotypes of mungbean after 70 days of sowing. –ve control (without pathogen, Zn or GM); +ve control [*M. phaseolina* (MP)]. Vertical bars show standard errors of means of three replicates. Means with the same letters are not significantly different at *p* < 0.05 according to Fisher’s least significant difference (LSD) test.

The growth attributes like shoot length were insignificantly affected; however, root length and biomass were significantly declined by 30–50% in the positive control of susceptible genotype (MNUYT-107) as compared to the negative control (healthy uninoculated plants). Soil application with GM alone did not improve the investigated attributes significantly, while 2% GM in combination with either level of Zn significantly enhanced all growth attributes up to 2-folds as compared to the positive control (Figures 2A–F). In the highly susceptible genotype (MNUYT-105), the pathogen stress in positive control treatments caused a significant reduction of 30–40% and 50–60% in length and biomass (fresh and dry) of both root and shoot over the negative control. Application of 2% GM only significantly improved the shoot attributes by 31%, but the same treatment did not improve root growth attributes significantly. However, all growth attributes improved significantly up to 2-folds in the combined application of 2% GM with medium or higher levels of Zn in MNUY-105 (Figures 2G–L).

### Biophysical yield attributes

**FIGURE 3:**
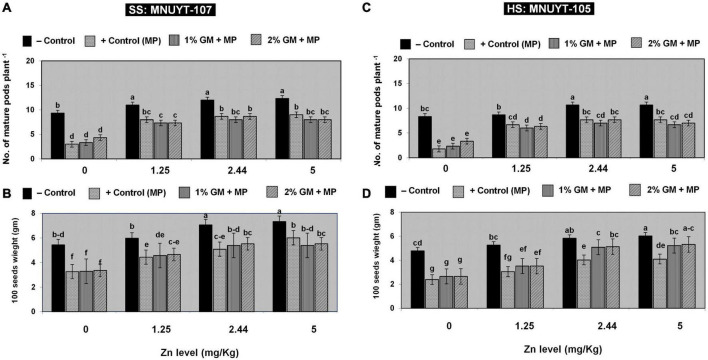
**(A–D)** Effect of soil application with zinc (Zn) and green manure (GM) on reproductive attributes in susceptible (SS: MNUYT-107) and highly susceptible (HS: MNUYT-105) genotypes of mungbean after 70 days of sowing. –ve control (without pathogen, Zn or GM); +ve control [*M. phaseolina* (MP)]. Vertical bars show standard errors of means of three replicates. Means with the same letters are not significantly different at *p* < 0.05 according to Fisher’s least significant difference (LSD) test.

The net effect of disease on vegetative attributes drastically decreased the number of matured pods, 100-seed weight, grain yield, biological yield, and harvest index per plant by 60–80% in susceptible and 70–90% in highly susceptible genotypes of mungbean. Like in vegetative growth assays, all yield-related parameters in both genotypes were more profoundly improved with a medium (2.44 mg/kg) or higher (5 mg/kg) dose of Zn combined with 2% GM (Figures 3A–D and Table 2).

**TABLE 2:** Zinc (Zn) and green manure (GM) impact on physiological attributes in susceptible mungbean genotypes (MNUYT-107) of mungbean inoculated with *Macrophomina phaseolina*(MP).

Parameters	Zn dose (mg/kg)	−Control	+ Control (MP)	GM + MP		Mean (F)
				1%	2%	
Total chlorophyll content (mg/g FW)	**0**	4.61 de	3.12 fg	3.81 e–g	3.91 e–g	**3.86 C**
	**1.25**	5.13 cd	4.66 de	3.79 e–g	4.15 ef	**4.43 B**
	**2.50**	5.67 a–c	4.89 d	4.11 d–f	4.57 d–f	**4.81 B**
	**5**	5.45 b–d	4.81 d	4.32 d–f	5.81 a	**5.10 A**
		
	**Mean (F)**	**5.22 A**	**4.37 BC**	**4.01 C**	**4.61 B**	

Carotenoids (mg/g FW)	**0**	2.89 b–d	1.61 i	1.78 g–i	2.31 e–g	**2.15 B**
	**1.25**	3.13 a–c	1.98 g–i	2.14 gh	2.56 c–e	**2.45 A**
	**2.50**	3.29 a–c	2.34 ef	2.21 gh	2.89 b–d	**2.68 A**
	**5**	3.55 a	2.55 c–e	2.15	3.15 ab	**2.82 A**
		
	**Mean (F)**	**3.22 A**	**2.12 C**	**2.07 C**	**2.73 B**	

Total protein content (mg/g FW)	**0**	65.11 i	55.17 k	55.19 k	60.12 j	**58.90 D**
	**1.25**	76.12 e	69.13 h	71.12 gh	80.18 de	**74.14 C**
	**2.50**	79.13 cd	74.35 fg	76.19 e	86.49 bc	**79.04 B**
	**5**	81.89 de	77.18 de	77.24 de	90.31 a	**81.66 A**
		
	**Mean (F)**	**75.56 B**	**68.96 C**	**69.94 C**	**79.28 A**	

Polyphenol oxidase activity (units/mg/g protein)	**0**	1.73 e–g	0.91 k	1.11 j	1.36 hi	**1.28 C**
	**1.25**	2.12 d	1.26 i	1.33 hi	1.87 ef	**1.65 B**
	**2.50**	2.36 bc	1.65	1.79 e–g	2.19 cd	**2.00 AB**
	**5**	2.45 b	1.87 ef	1.91 e	2.54 a	**2.19 A**
		
	**Mean (F)**	**2.17 A**	**1.42 C**	**1.54 BC**	**1.99 B**	

Catalase activity (units/mg/g protein)	**0**	16.71 g	18.91 fg	23.22 de	25.13 b–d	**20.99 B**
	**1.25**	20.19 e	25.16 b–d	25.19 b–d	29.16 a	**24.43 A**
	**2.50**	21.93 e	25.67 b–d	25.16 b–d	27.91 a–c	**24.42 A**
	**5**	20.91 e	25.21 b–d	24.13 b–d	26.31 bc	**23.64 A**
		
	**Mean (F)**	**18.19 C**	**23.74 B**	**24.43 B**	**27.13 A**	

Superoxide dismutase activity (units/mg/g protein)	**0**	0.48 b–d	0.27 g	0.33 fg	0.35 e–g	**0.36 C**
	**1.25**	0.45 b–d	0.37 c–f	0.37 ef	0.41 cd	**0.40 B**
	**2.50**	0.54 a	0.38 c–f	0.41 cd	0.42 b–d	**0.44 A**
	**5**	0.51 a–d	0.41 b–d	0.38	0.43 b–d	**0.43 A**
		
	**Mean (F)**	**0.50 A**	**0.36 C**	**0.37 BC**	**0.40 B**	

Peroxidase activity (units/mg/g protein)	**0**	7.89 g	9.11 f–h	10.15 f	12.13 e	**9.82 B**
	**1.25**	8.91 gh	12.56 e	13.23 de	14.67 c	**12.34 A**
	**2.50**	8.11 fg	13.13 de	14.23 cd	15.89 b	**12.84 A**
	**5**	8.25 gh	13.29 de	14.28 cd	16.04 a	**12.97 A**
		
	**Mean (F)**	**8.29 C**	**12.02 B**	**12.97 B**	**14.68 A**	

Mean values with the same lower and upper case indicate insignificant difference (*p* ≤ 0.05) as determined by LSD Test.

### Biochemical attributes

#### Photosynthetic pigments

Photosynthetic pigments (total chlorophyll content and carotenoids) were significantly decreased by 30 and 50% in susceptible and highly susceptible genotypes, respectively, as compared to negative control, while GM, Zn, and their combinations variably improved the photosynthetic pigments by 30–70% as compared to positive control. Mean values (upper case) in the row showed the maximum total chlorophyll content and carotenoids were obtained due to the interactive effect of 5 mg/kg of Zn combined with 2% GM followed by 2.44 mg/kg combined with 2% GM (Tables 3, 4).

**TABLE 3:** Zinc (Zn) and green manure (GM) impact on physiological attributes in highly susceptible (MNUYT-105) genotypes of mungbean inoculated with *Macrophomina phaseolina*(MP).

Parameters	Zn dose (mg/kg)	−Control	+ Control (MP)	GM + MP		Mean (F)
				1%	2%	
Total chlorophyll content (mg/g FW)	**0**	4.26 e–g	2.89 j	3.81 f–h	3.91 fg	**3.72 B**
	**1.25**	4.39 ef	4.38 ef	3.19 i	3.74 f–h	**3.93 B**
	**2.50**	5.13 b–d	4.67 c–e	3.48 hi	4.63 c–e	**4.48 AB**
	**5**	4.78 cd	4.69 c–e	4.05 f	5.91 a	**4.86 A**
		
	**Mean (F)**	**4.64 A**	**4.16 B**	**3.63 C**	**4.55 AB**	

Carotenoids (mg/g FW)	**0**	2.39 b–d	1.21 i	1.92 e–g	2.26 cd	**2.39 A**
	**1.25**	1.84 fg	1.74 gh	1.23 i	1.63	**1.84 B**
	**2.50**	2.37 b–d	2.11 c–e	1.48	2.26 cd	**2.37 A**
	**5**	2.12 c–e	2.18 c–e	1.98 ef	2.90 a	**2.12 A**
		
	**Mean (F)**	**2.18 A**	**1.81 B**	**1.65 B**	**2.26 A**	

Total protein content (mg/g*FW)	**0**	60.11 e	32.10 k	45.12 j	47.19 ij	**46.08 C**
	**1.25**	70.31 d	50.23 h	44.13 j	60.22 e	**56.03 B**
	**2.50**	90.34 a	59.76 ef	51.12 gh	83.56 bc	**70.78 A**
	**5**	80.15 c	60.31 e	54.67 f	89.81 a	**70.92 A**
		
	**Mean (F)**	**75.00 A**	**50.25 C**	**48.76 C**	**69.80 B**	

Polyphenol oxidase activity (units/mg/g protein)	**0**	1.73 e	0.55 l	0.67 k	0.81 j	**0.94 D**
	**1.25**	1.79	1.01	0.97 ij	1.35 f–h	**1.28 C**
	**2.50**	1.85 bc	1.19 hi	1.24 g–i	1.78 c–e	**1.52 B**
	**5**	1.79 cd	1.25 gh	1.31 gh	1.89 a	**1.56 A**
		
	**Mean (F)**	**1.79 A**	**1.11 C**	**1.05 C**	**1.46 B**	

Catalase activity (units/mg/g protein)	**0**	0.43 c–e	0.23 j	0.24 j	0.27 i	**0.29 C**
	**1.25**	0.42 de	0.37 fg	0.27 i	0.43 cd	**0.37 B**
	**2.50**	0.53 a	0.39 fg	0.31 h	0.44 cd	**0.41 A**
	**5**	0.47 b–d	0.41 ef	0.36 g	0.44 cd	**0.42 A**
		
	**Mean (F)**	**0.46 A**	**0.35 C1**	**0.30 D**	**0.40 B**	

Superoxide dismutase activity (units/mg/g protein)	**0**	17.92 bc	9.19 h	12.45 fg	13.13 e–g	**13.17 C**
	**1.25**	21.32 a	15.15 c–e	11.15	16.81 cd	**16.10 B**
	**2.50**	19.11 a–c	16.31 cd	14.56 ef	18.35 bc	**17.06 A**
	**5**	18.62 a–c	17.16 b–d	15.17 c–e	20.67 ab	**17.90 A**
		
	**Mean (F)**	**19.20 A**	**14.45 C**	**13.33 D**	**17.24 B**	

Peroxidase activity (units/mg/g protein)	**0**	8.34 de	4.52 j	4.11 j	6.45 hi	**5.86 C**
	**1.25**	11.73 a	6.89 g–i	5.57 i	7.97 e–g	**8.04 A**
	**2.50**	9.35 b–d	7.14 fg	6.98 gh	8.13 de	**7.90 AB**
	**5**	8.25 de	7.45 e–g	7.12 fg	8.57 cd	**7.85 B**
		
	**Mean (F)**	**9.42 A**	**6.50 C**	**5.95 D**	**7.78 B**	

Mean values with the same lower and upper case indicate insignificant difference (*p* ≤ 0.05) as determined by LSD Test.

**TABLE 4:** Impingement of different levels of zinc (Zn) and green manure (GM) on harvest index, grain and biological yield in susceptible (SS: MNUYT-107) and highly susceptible (MNUYT-105) genotypes of mungbean.

Treatments	Grain yield/plant			Biological yield/plant			Harvest index (%)		
	SS	HS	Mean	SS	HS	Mean	SS	HS	Mean
T_1_	4.56	3.59	**4.07**	14.33	13.27	**13.80**	31.74	26.96	**29.35**
T_2_	0.88	0.39	**0.64**	6.16	4.57	**5.36**	13.39	8.34	**10.87**
T_3_	0.99	0.56	**0.77**	7.33	5.80	**6.57**	14.30	9.66	**11.98**
T_4_	1.30	0.80	**1.05**	7.69	6.09	**6.89**	17.13	13.51	**15.32**
T_5_	5.91	4.10	**5.01**	17.82	14.93	**16.37**	33.10	27.41	**30.25**
T_6_	3.20	1.83	**2.52**	12.93	10.48	**11.71**	24.74	17.62	**21.18**
T_7_	3.01	1.90	**2.46**	10.51	8.30	**9.41**	28.82	23.09	**25.96**
T_8_	3.07	2.00	**2.54**	10.61	8.44	**9.53**	28.96	23.85	**26.41**
T_9_	7.62	5.59	**6.61**	19.59	16.48	**18.04**	38.79	33.95	**36.37**
T_10_	3.96	2.77	**3.37**	14.78	12.50	**13.64**	26.71	22.12	**24.41**
T_11_	3.88	3.20	**3.54**	12.48	10.70	**11.59**	31.14	29.94	**30.54**
T_12_	4.31	3.53	**3.92**	14.02	12.14	**13.08**	30.83	29.26	**30.05**
T_13_	8.18	5.82	**7.00**	20.16	16.72	**18.44**	40.37	34.66	**37.52**
T_14_	4.87	2.82	**3.85**	15.73	12.59	**14.16**	30.97	22.55	**26.76**
T_15_	3.89	3.13	**3.51**	13.59	11.74	**12.67**	28.63	26.84	**27.74**
T_16_	3.98	3.35	**3.66**	13.68	11.96	**12.82**	29.02	27.97	**28.50**

T_1_, Negative control; T_2_, Positive control [M. phaseolina(MP)]; T_3_, 1% GM + MP; T_4_, 2% GM + MP; T_5_, Zn (1.25 mg/kg); T_6_, Zn (1.25 mg/kg) + MP; T_7_, 1% GM + MP + Zn (1.25 mg/kg); T_8_, 2% GM + MP + Zn (1.25 mg/kg); T_9_, Zn (2.44 mg/kg); T_10_, Zn (2.44 mg/kg) + MP; T_11_, 1% GM + MP + Zn (2.44 mg/kg); T_12_, 2% GM + MP + Zn (2.44 mg/kg); T_13_, Zn (5 mg/kg); T_14_, Zn (5 mg/kg) + MP; T_15_, 1% GM + MP +Zn (5 mg/kg); T_16_, 2% GM + MP + Zn (5 mg/kg). Bold letters indicate the mean value of yield attribute of two genotyped of mungbean.

#### Antioxidant enzymes

In positive control of susceptible genotype, TPP, CAT, and POX were affected insignificantly, while SOD and PPO decreased by 40–50% as compared to negative control. Zn (1.25–5 mg/kg) alone and in combination with 1% GM statistically exhibited an equal effect on the said biochemical traits. Therefore, PPO activity was significantly raised by 100%, and the remaining biochemical traits (TPP, SOD, CAT, and POX) increased by 30–60% over the positive control. Moreover, all-biochemical traits of susceptible genotypes were affected enormously by 50–80% in Zn (1.25–5 mg/kg) + 2% GM treatments, except PPO, which improved up to 200% as compared to the positive control (Table 3). In the highly susceptible genotype, all biochemical traits declined by 50%, though PPO was reduced more drastically by 70% as compared to its corresponding negative control. Despite this, Zn only significantly improved TPP and PPO by up to 100% and antioxidants by 40–60%. Nevertheless, Zn + 2% GM enhanced the TPP and PPO up to 200%, and antioxidants up to 100% in highly susceptible genotypes as compared to their corresponding positive controls (Table 4).

Pairwise comparisons with bold letters in rows and columns in Tables 3, 4 indicate the individual and bilateral effects of Zn and GM. Accordingly, Zn only was found to be a better treatment than GM only for improving biochemical traits in susceptible and highly susceptible genotypes of the mungbean. Moreover, Zn (2.44 and 5 mg/kg) along with 2% GM (bold letters in rows) were found as the most effective treatments for boosting all biochemical traits in both genotypes of mungbean growing under the stress of *M. phaseolina* (Tables 3, 4).

#### Multivariate analysis

**FIGURE 4:**
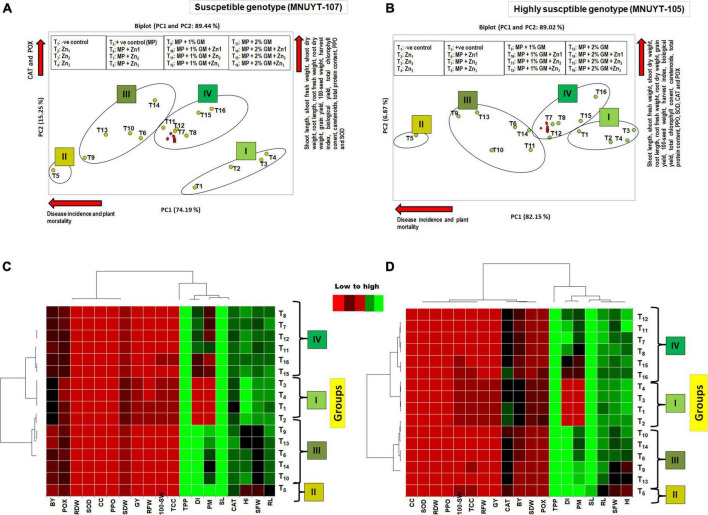
**(A–D)** Principal component analysis and heatmap of disease pressure, biophysical, biochemical traits of mungbean genotypes due to the effect of *Macrophomina phaseolina* (MP), zinc (Zn), and green manure (GM) in pots bioassays. DI, disease incidence; PM, plant mortality; SL, shoot length; SFW, shoot fresh weight; SDW, shoot dry weight; RL, root length; RFW, root fresh weight; RDW, root dry weight; SW, 100 seed-weight; GY, grain yield; BY, biological yield; HI, harvest index; TCC, total chlorophyll content; CC, carotenoids; TPC, total protein content; SOD, superoxide dismutase; CAT, catalase; POX, peroxidase; PPO, polyphenol oxidase. In PCA diagram, the green circle represents treatments, and the red circle represents attributes. T_1_, –ve control; T_2_, Zn_1_ (1.25 mg/kg); T_3_, Zn_2_ (2.44 mg/kg); T_4_, Zn_3_ (5 mg/kg); T_5_, +ve control [*M. phaseolina* (MP)]; T_6_, Zn_1_ (1.25 mg/kg) + MP; T_7_, Zn_2_ (2.44 mg/kg) + MP; T_8_, Zn_3_ (5 mg/kg) + MP; T_9_, 1% GM + MP; T_10_, 1% GM + MP + Zn_1_ (1.25 mg/kg); T_11_, 1% GM + MP + Zn_2_ (2.44 mg/kg); T_12_, 1% GM + MP + Zn_3_ (5 mg/kg); T_13_, 2% GM + MP; T_14_, 2% GM + MP + Zn_1_ (1.25 mg/kg); T_15_, 2% GM + MP + Zn_2_ (2.44 mg/kg); T_16_, 2% GM + MP + Zn_3_ (5 mg/kg).

Biplot-based PCA and heatmap analyses of 19 different biophysical and biochemical traits of both genotypes of mungbean were used as an additional comparison between 16 different treatments. Both analyses clearly separated the pathogen-inoculated treatments from the uninoculated treatments. PCA explained 89% of the total variation by the first two components (Figures 4A,B), and the resulting four large groups coincided with those of the heatmap (Figures 4C,D). Group I comprised all four treatments not inoculated with the pathogen (T_1_–T_4_); the remaining 12 treatments (T_6_–T_16_) provided with the pathogen were segregated into groups II, III, and IV. Treatment/s in group II containing positive control (T_5_) was the lowest for all evaluated traits. Groups III and IV comprised treatments, provided with Zn, GM, and their combinations, which showed an appreciable effect of Zn and GM on the investigated traits. The treatments mostly consisted of Zn (2.44 or 5 mg/kg) + 2% GM in group IV, more toward the right side of the biplot and near to negative control (T_1_), were the highest for biophysical and biochemical traits along with minimum to negligible disease. Moreover, all biophysical and biochemical traits in similar directions indicated positive correlation among themselves, while these were negatively correlated with the disease (disease incidence and plant mortality) in both genotypes.

## Discussion

Charcoal rot diseases in mungbean are yield-limiting problems in arid and water-deficient regions. In mungbean growing areas (Bhakkar, Khushab, Mianwali, Layyah, and Muzzaffargarh) of Pakistan, climatic conditions (high temperate, dry, and desert conditions) and soil (calcareous and nutrient-deficient soil) encourage the prevalence of *M. phaseolina*. Since Zn is essential for the growth and reproduction of plants, basal applications of Zn fertilizers combined with green manures could be effective in improving crop productivity by mitigating charcoal rot disease stress in mungbean (Khan et al., 2018).

**FIGURE 5:**
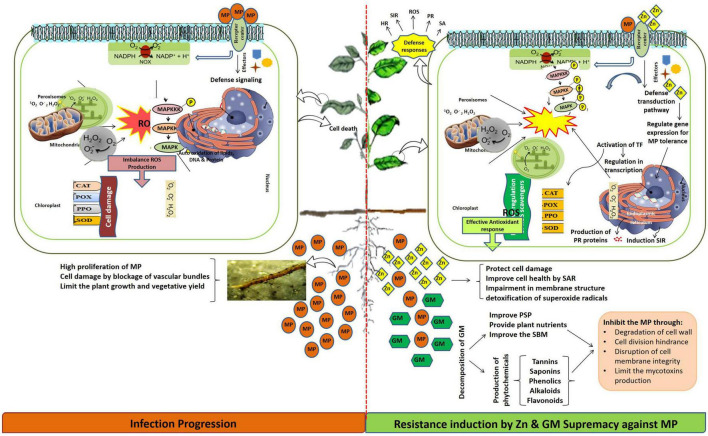
Possible effect of *Macrophomina phaseolina* (MP), zinc (Zn), and green manure (GM) in mungbean genotypes.

In this study, disease pressure incited by *M. phaseolina* caused a significant reduction in morpho-growth, physicochemical, and yield-related attributes in the positive control of highly susceptible (MNUYT-105) and susceptible (MNUYT-107) genotypes of mungbean by 30–90% and 30–80%, respectively. The final fate of the plant up to 70 days under pathogen stress appeared as stunted growth; shattering of leaves and flowers; dry, immature, and empty pods; and small grains that turned to white-gray color and finally deformed (Fuhlbohm et al., 2013; Khan et al., 2016). The growth of *M. phaseolina* might have caused clogging of the vascular system, which can easily disrupt host cell defensive machinery through over-accumulation of ROS, production of toxins, and cell wall degrading enzymes (Marquez et al., 2021), hence altered biochemical attributes (TCC, CC, TPP, PPO, SOD, CAT, and POX), which ultimately decreased biophysical and yield-related attributes in the mungbean genotypes (Awan et al., 2019). It is predicted that the pathogen might be undetected (highly susceptible genotype) or detected late (susceptible genotype), which has caused circumventing or hijacking of the plant self-defense system (Awan et al., 2019). Accordingly, disturbance in electron flow at the mitochondria and chloroplast membranes through overproduction of ROS may decrease water uptake, increase CO_2_ concentration and stomatal closure, and may lead to cell death by pathogen intrusion inside cells (Figure 5). Therefore, the establishment of *M. phaseolina* through systemic infection deteriorated seeds as observed through minimum yield-related traits in mungbean genotypes, while the infected seeds might be responsible for the introduction of the pathogen into new production areas (Fuhlbohm et al., 2013).

The mungbean plants grown in soil supplied with Zn fertilization (1.25–5 mg/kg) alone and in combination with GM were protected against *M. phaseolina* attack, and this protection was observed as reduced disease incidence and plant mortality in both genotypes. The alleviation of disease stress was accompanied by improvement in biophysical (50–100%) and biochemical (30–100%) attributes, which sustainably enhanced grain average yield (300–600%), biological yield (100–200%), and harvest index (100–200%) in both genotypes. Either medium (2.44 mg/kg) or higher (5 mg/kg) levels of Zn fertilizer combined with 2% GM exhibited the highest benefits on the investigated attributes of the mungbean plants. Added liquid forms of Zn in Zn-deficient soil in the arid and semi-arid regions were likely to remain near the surface and might be frequently available for uptake and utilization by the plants. Generally, Zn can easily diffuse from the root to the xylem through specific transporters in the plasma membrane, then toward shoots in the transpiration stream. Hence, either in the soil or inside a plant, Zn might act directly against the pathogen by avoiding conidial production and pathogen reproduction in soil (Luo et al., 2020), which may cause systemic acquired tolerance due to a decrease in the pathogen population (Quaglia et al., 2021). Martos et al. (2016) findings attributed the dual effect of Zn as a direct inhibitor of fungal pathogen and potential enhancer of fungal-induced systemic resistance by jasmonic acid. Moreover, inside plant Zn probably contributes to the plant physiological pathways, where it can activate and regularize the function of the enzymatic defense mechanisms associated with detoxification of ROS, metabolism of macromolecules (proteins, nucleic acid, and carbohydrates), callose apposition and auxin synthesis (Sadeghzadeh, 2013). Therefore, Zn, possibly by ensuring structural and functional integrity of the membrane and by inducing systemic resistance in the mungbean plant, assisted in the suppression of charcoal rot disease and improvement in morpho-physiological and yield-related traits (Khan et al., 2018; Shoaib et al., 2020, 2022). Finally, a reduction in the disease pressure may result in greater photosynthetic pigment and more production of effective antioxidant defense enzymes (e.g., SOD, CAT, POX, and PPO) in host plants. Mamnabi et al. (2020) linked the activities of SOD, CAT, POX, and PPO with the increase in grain yield of wheat under drought stress after the application of biofertilizer combined with chemical fertilizer. Wang et al. (2019) suggested the role of the acid-mediated oxidative burst in wheat against stripe rust disease due to the action of Zn-binding protein. Therefore, in this study, the dual function of Zn might be predicted in simultaneously limiting the pathogen attack by its antifungal action and activating the mungbean plant defense system.

Under combined application of Zn + GM, a better outcome in disease suppression could be attributed to the benefit of decomposition of GM (*S. bispinosa*), which releases the organic acid in the soil, lowers soil pH, improves soil physicochemical properties, and increases the availability of Zn and essential nutrients to the plant (De et al., 2021). Moreover, the multivariate analysis also separated the best treatments for the disease management and suggested that Zn (2.44 or 5 mg/kg) in combination with GM may act in the way that ideally managed charcoal rot disease in mungbean plants by regulating plant development and activities of antioxidants and increasing grain yield.

Moreover, the application of GM (1 or 2%) only exhibited the least benefits on the crop attributes as compared to other treatments possibly due to the incorporation of high amounts of readily degradable organic matter, which might increase the competition for oxygen in the soil, caused by the resulting intense macro and microbial activity. Besides, GM also releases different types of acids that may cause seed dormancy and can damage new seedlings. Therefore, planting and especially sowing of a new crop immediately following the incorporation of the green manure should be avoided.

## Conclusion

Integrative crop protection with the precision application of Zn (2.44 or 5 mg/kg) combined with 2% green manure (*S. bispinosa*) has been eco-friendly and cost-effective in enhancing mungbean yield while suppressing charcoal rot disease. Such a management strategy should be applied to combat charcoal rot disease, especially in Zn-deficient soil.

## Data availability statement

The raw data supporting the conclusions of this article will be made available by the authors, without undue reservation.

## Author contributions

AS: conceptualization, methodology, formal analysis, writing—original draft preparation, review and editing, supervision, and project administration. KK: conceptualization, methodology, data curation, formal analysis, and editing. ZA: data curation. BJ and PK: revision, data analysis, and language editing. All authors have read and agreed to the published version of the manuscript.
